# The Current State of Realistic Heart Models for Disease Modelling and Cardiotoxicity

**DOI:** 10.3390/ijms25179186

**Published:** 2024-08-24

**Authors:** Kornél Kistamás, Federica Lamberto, Raminta Vaiciuleviciute, Filipa Leal, Suchitra Muenthaisong, Luis Marte, Paula Subías-Beltrán, Aidas Alaburda, Dina N. Arvanitis, Melinda Zana, Pedro F. Costa, Eiva Bernotiene, Christian Bergaud, András Dinnyés

**Affiliations:** 1 BioTalentum Ltd., Aulich Lajos Str 26, H-2100 Gödöllő, Hungary; kornel.kistamas@biotalentum.hu (K.K.); suchitra.polgari@biotalentum.hu (S.M.); melinda.zana@biotalentum.hu (M.Z.); 2 Department of Physiology and Animal Health, Institute of Physiology and Animal Nutrition, Hungarian University of Agriculture and Life Sciences, Páter Károly Str 1, H-2100 Gödöllő, Hungary; 3 Department of Regenerative Medicine, State Research Institute Innovative Medicine Centre, Santariskiu g. 5, LT-08406 Vilnius, Lithuania; raminta.vaiciuleviciute@imcentras.lt (R.V.); aidas.alaburda@gf.vu.lt (A.A.); eiva.bernotiene@imcentras.lt (E.B.); 4 Biofabics Lda, Rua Alfredo Allen 455, 4200-135 Porto, Portugalpedro.costa@biofabics.com (P.F.C.); 5 Digital Health Unit, Eurecat—Centre Tecnològic de Catalunya, 08005 Barcelona, Spain; luis.marte@eurecat.org (L.M.); paula.subias@eurecat.org (P.S.-B.); 6 Institute of Biosciences, Life Sciences Center, Vilnius University, Sauletekio al. 7, LT-10257 Vilnius, Lithuania; 7 Laboratory for Analysis and Architecture of Systems—French National Centre for Scientific Research (LAAS-CNRS), 7 Avenue du Colonel Roche, F-31400 Toulouse, France; constandina.arvanitis@laas.fr (D.N.A.); bergaud@laas.fr (C.B.); 8 Faculty of Fundamental Sciences, Vilnius Tech, Sauletekio al. 11, LT-10223 Vilnius, Lithuania

**Keywords:** hiPSC-CM, cardiomyocyte, toxicology, drug testing, cardiomyocyte maturation, heart-on-a-chip, cardiac model, disease modelling

## Abstract

One of the many unresolved obstacles in the field of cardiovascular research is an uncompromising in vitro cardiac model. While primary cell sources from animal models offer both advantages and disadvantages, efforts over the past half-century have aimed to reduce their use. Additionally, obtaining a sufficient quantity of human primary cardiomyocytes faces ethical and legal challenges. As the practically unlimited source of human cardiomyocytes from induced pluripotent stem cells (hiPSC-CM) is now mostly resolved, there are great efforts to improve their quality and applicability by overcoming their intrinsic limitations. The greatest bottleneck in the field is the in vitro ageing of hiPSC-CMs to reach a maturity status that closely resembles that of the adult heart, thereby allowing for more appropriate drug developmental procedures as there is a clear correlation between ageing and developing cardiovascular diseases. Here, we review the current state-of-the-art techniques in the most realistic heart models used in disease modelling and toxicity evaluations from hiPSC-CM maturation through heart-on-a-chip platforms and in silico models to the in vitro models of certain cardiovascular diseases.

## 1. Introduction

Cardiovascular diseases (CVDs) are the most prevalent diseases and the leading cause of death worldwide. In recent decades, most of the phase 3 cardiac clinical trials failed due to safety concerns, lack of efficiency, or economic considerations as we described previously [1]. This was best illustrated by the disappointing outcomes of the Cardiac Arrhythmia Suppression Trial (CAST), CAST II, and Survival with oral d-sotalol (SWORD) trials. In these studies, antiarrhythmic drugs (encainide, flecainide, moricizine, or d-sotalol) were administered; however, in the CAST trial, the number of deaths due to arrhythmia or shock after recurrent myocardial infarction was significantly higher in the treated patient groups [2], while the CAST II and SWORD trials resulted in higher risk of drug-associated mortality (cardiac arrests due to arrhythmias) in patients assigned to the drug treatment [3,4,5]. Unexpected adverse effects (e.g., hidden cardiotoxicity), therefore, will lead to the discontinuation of clinical studies and withdrawal of the drug from the market, leading to the failure of new therapies and an immense financial burden [6].

As a consequence of the unsuccessful clinical attempts, only nine drugs targeting CVDs were approved by the U.S. Food and Drug Administration (FDA) in the last decade [1]. To date, ticagrelor was approved in 2011 to reduce cardiovascular death and heart attack in patients with acute coronary syndromes [7]; vorapaxar in 2014 to reduce the risk of heart attacks and stroke in high-risk patients [8]; ivabradine and sacubitril/valsartan in 2015 to reduce hospitalisation from worsening heart failure [9] and to treat heart failure [10], respectively; tafamidis meglumine in 2019 to treat cardiomyopathy caused by transthyretin-mediated amyloidosis in adults [11]; vericiguat in 2021 to mitigate the risk of cardiovascular death and hospitalisation for chronic heart failure [12]; mavacamten in 2022 to cure patients with obstructive hypertrophic cardiomyopathy [13]; sotagliflozin in 2023 to treat heart failure [14]; and aprocitentan in 2024 to treat resistant hypertension [15].

The development of realistic cardiac models for preclinical drug testing and cardiotoxicity assessment could significantly reduce the above-mentioned failure rates in clinical trials by effectively addressing safety concerns. Furthermore, these advanced models have the potential to enhance drug development, leading to the production of more effective treatments for cardiovascular diseases. There is an urgent need for novel and more realistic heart models that could both replace animal research in preclinical studies and serve as tools of human origin to better identify drug candidates that can reach production and large-scale treatment of patients. This toolset will not only allow the selection of potential drug molecules but can save a tremendous amount of time and expense by identifying adverse drug effects as early risk prediction before the clinical studies. Recently, human induced pluripotent stem cell-derived cardiomyocytes (hiPSC-CMs) have been considered as potential options for in vitro cytotoxicity evaluations, high-throughput drug screenings, and complex systems, such as organ-on-a-chip models. Besides assessing the safety profiles of a compound in the preclinical phase, hiPSC-CMs are anticipated to serve as an ideal cellular source for regenerative and precision or personalised medicine. Ongoing clinical trials attempt, for example, implanting clinical-grade hiPSC-CMs as surgical grafts to treat patients [16] or directly injecting allogeneic hiPSC-CM spheroids to remuscularise the injured myocardium [17]. The hiPSC-CMs have also been considered as valuable resources for modelling certain genetic heart diseases such as long QT syndrome (LQTS) and cardiomyopathies (hypertrophic or dilated cardiomyopathy and arrhythmogenic cardiomyopathy) or atrial fibrillation (AF), enabling the study of their molecular pathologies [18]. On the other hand, the maturity status of hiPSC-CMs is still a main bottleneck in the field. To better understand the real pathophysiology, especially considering the often late in-life onset of cardiac diseases, and to translate these findings into improved drug development and more evolved disease modelling, it is key to pay special attention to pioneering maturation studies. In this review, we summarise the current state-of-the-art in the field of hiPSC-CMs, focusing on the maturation level of these cells also from a bioengineering point of view, while highlighting the in silico and disease modelling applications.

## 2. The Potential of hiPSC-CMs and the Substantial Issues with Maturation

Reprogramming of somatic cells to a pluripotent state was a groundbreaking discovery that led to the generation of human induced pluripotent stem cells (hiPSCs) [19]. The pluripotency of hiPSCs offers the possibility of creating virtually every cell type of the human body through directed differentiation protocols. Soon after the creation of hiPSCs, the first publications were released on the generation of hiPSC-CMs. The iPSC technology and, moreover, the possible applications of human-based cardiac models are expected to revolutionise and transform the field and regenerative medicine. Heart models in research and in drug screening take advantage of the unlimited human cardiomyocytes (CMs) without the ethical debates over the application of embryonic stem cells (ESCs) or without the translational challenges of animal models.

**Figure 1:**
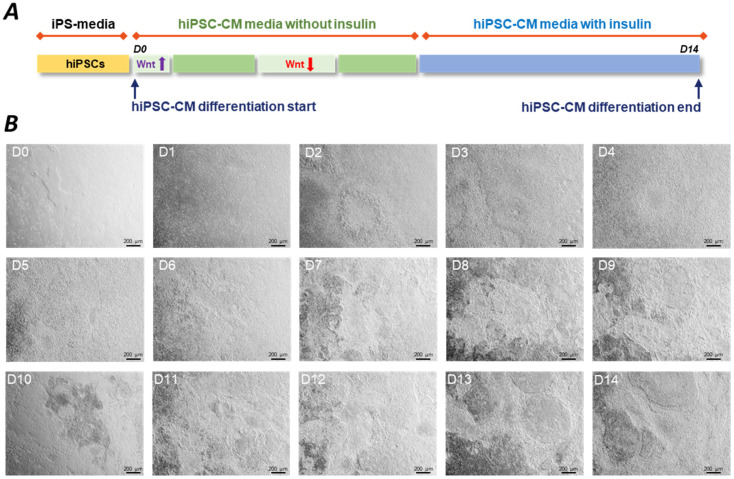
The process of hiPSC-CM differentiation. (**A**) Conventional cardiac differentiation protocols use CHIR99021 and IWP-2 or IWR-1 small molecules to sequentially activate and inhibit the Wnt/β-catenin pathway, respectively. This panel shows a representative example of a typically applied hiPSC-CM differentiation protocol. (**B**) Representative images show the process of cardiac induction from Day 1 hiPSCs (D1) to Day 14 (D14) beating hiPSC-CMs. Small molecules were applied to a confluent hiPSC culture on D0. Cardiomyocytes showed spontaneous beating from D7. At D14, harvesting of the hiPSC-CMs is followed by functional readouts and cryopreservation. Images were taken on hiPSC-CMs generated by BioTalentum Ltd., at 4× magnification; scale bar, 200 μm.

**Figure 2:**
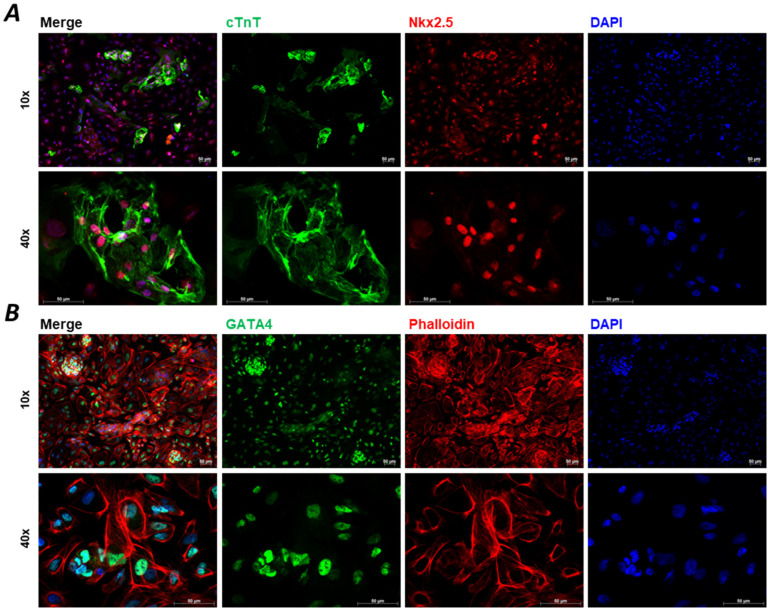
Immunocytochemistry staining of D15 2D hiPSC-CMs. Immunocytochemistry images of specific biomarkers for hiPSC-CM characterisation at Day 15 (D15). Cardiac troponin T (cTnT) is the most generally used marker for CMs, which shows apparent striations ((**A**), green) in the case of the development of CM sarcomere structures. Other biomarkers, such as mesodermal marker homeobox protein Nkx2.5 ((**A**), red), transcription factor GATA4 ((**B**), green), and phalloidin ((**B**), red) are shown, while 4′,6-diamidino-2-phenylindole (DAPI) shows nuclear staining ((**A**,**B**), blue). Images were taken on hiPSC-CMs generated by BioTalentum Ltd., at 10× and 40× magnification; scale bar, 50 μm.

The de novo CMs are generated by cardiac differentiation. The cardiac induction is initiated on the hiPSC cultures and can be performed in both 2D and 3D arrangements. Early attempts to produce CMs followed the hanging drop culture technique, while later studies applied 2D plate formats or low attachment plates for 3D aggregates. There is also evidence that co-culture with other cell types can help the survival of hiPSC-CMs. The different approaches of CM differentiation were summarised elsewhere [1,20]. In general, several protocols follow a 14-day cultivation with the application of small molecules or growth factors or alternatively, a combination of the two (Figure 1) [1]. These techniques allow researchers to collect viable beating cells at Day 14 (D14) with around 70–90% of the cell population positive to cardiac troponin T (cTnT), a clear marker of CM fate (Figure 2).

Allogeneic or autologous cell transplantation paved the way for cardiac personalised and regenerative medicine already with several clinical trials in diseases such as ischaemic cardiomyopathy and heart failure [16,21,22,23,24,25,26]. These clinical-grade hiPSC-CMs are transplanted into patients following cardiac differentiation, where the purity of the culture is key. Undoubtedly, superior CM maturation can be observed in vivo and besides the numerous factors that drive this process, time is also a crucial aspect of CM maturation [27]. While a study in guinea pigs showed reduced arrhythmia incidence after transplanting human ESC-derived CMs (hESC-CMs) [28], transplanted de novo hESC-CMs in non-human primate hearts or allogeneic iPSC-CMs in cynomolgus hearts might carry the risk of post-transplant arrhythmogenicity with idioventricular rhythms or even with sustained ventricular tachycardia, respectively [29,30].

**Figure 3:**
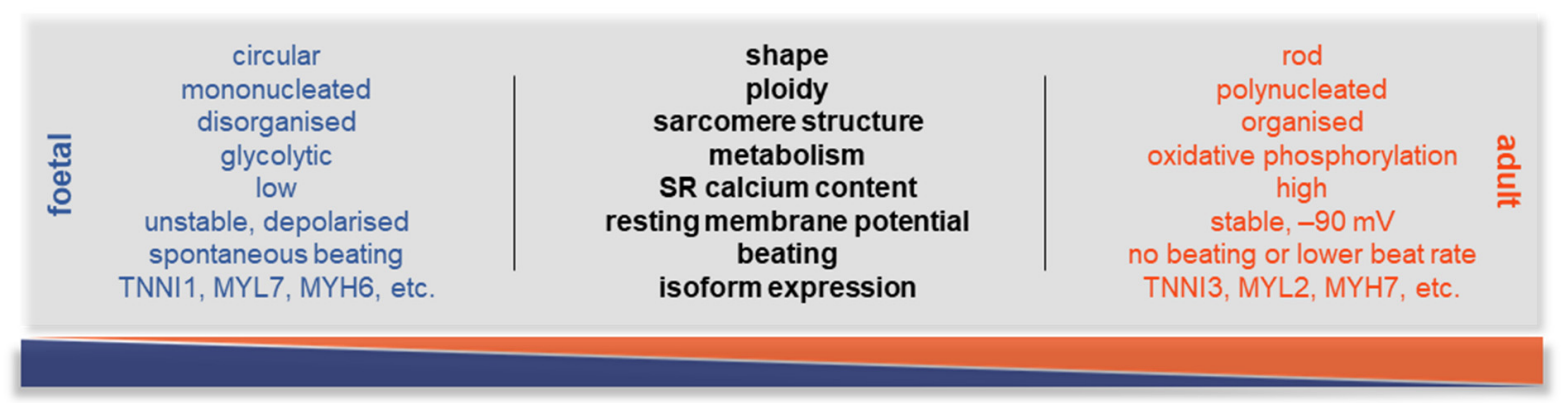
Key differences between immature and mature CMs. Summary of the main physiological parameters that are different in a foetal CM compared to an adult one. During maturation, many processes evolve, from the shape of the cells to the metabolic switch. One of the most important molecular changes is the development of the adult ion channel pool and the adult-like electrophysiologic properties, allowing a stable resting membrane potential and, therefore, a physiological AP and non-spontaneous contractions.

On the other hand, those hiPSC-CMs that are used for in vitro drug testing and heart-on-a-chip (HOC) platforms in healthy conditions and disease modelling lack a pivotal element: the maturation of the cells. Using existing protocols, hiPSC-CMs are developmentally immature, with foetal-like CM properties, such as glycolytic metabolism, circular cellular shape, lack of sarcomere alignment, and expression of foetal genes in their sarcomere components (e.g., TNNI1 rather than TNNI3). On the other hand, mature CMs express a different set of maturation markers (e.g., MYH7, TNNI3, connexin-43, and TTNN2B), while characterised by a distinct morphology, including hypertrophic, rod-shaped cellular body, higher myofibril density and alignment, and polyploidy (Figure 3). There are also characteristic differences in their electrophysiology; during maturation, the unstable membrane potential—constant diastolic depolarisation leading to spontaneous activity—changes to a stable resting membrane potential at around −80–90 mV.

**Figure 4:**
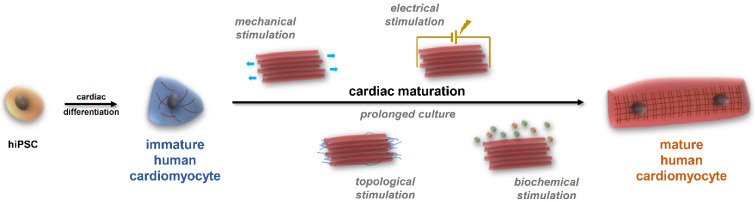
The multifactorial process of cardiac maturation. HiPSCs can differentiate into virtually all cell types. To produce beating muscle cells during cardiac differentiation, small molecules are applied; however, these cells usually do not reach the desired maturity status. To achieve maturation, several approaches are available—besides the time factor—in biomaterial sciences, including various stimuli (mechanical, electrical, topological, and biochemical stimulation, detailed in the text).

Although the currently available protocols give a high yield of viable cells, the usually applied 2–3 weeks of cardiac differentiation is not sufficient to reach an adult phenotype in these cells (Figure 2). Additionally, it has been shown that several factors are needed to age hiPSC-CMs (Figure 4) [1]. In the drug development pipeline and disease modelling, maturity of the cells is crucial as in most of the cases the older the patient, the higher the likelihood of developing CVDs, the more essential it is to reach an adult phenotype in drug testing platforms [31]. Several approaches have been tested in recent years to overcome this developmental stalemate, but there is no fully adequate answer yet to this unresolved scientific question [32]. On the other hand, it must be noted that the full potential of early hiPSC-CMs can be exploited if the overall goal is to study neonatal diseases or to develop neonatal disease models and drug screening platforms and establish methods for developmental toxicity [33,34,35].

### 2.1. Standardisation Procedures

Despite their immense potential, harnessing hiPSC-CMs for research and therapeutic purposes needs to overcome several challenges. A primary challenge lies in the necessity to establish standardised procedures for the differentiation process, aiming to assure reproducibility and consistency across various experiments and laboratory settings. A significant achievement in standardisation efforts has been defined through the use of chemically defined protocols for generating functional hiPSC-CMs [36,37]. Nonetheless, in hiPSC-CM cultures, the presence of endothelial cells and fibroblasts alongside CMs underscores the inherent cellular heterogeneity, while further complexity is introduced by the differential expression of CM subtypes, with distinct characteristics observed between atrial and ventricular populations [28,38,39]. This cellular heterogeneity is a prominent feature in these cultures and it is known that factors such as substrate rigidity, the duration and dosage of growth factors, or oxygen levels in the culture milieu contribute substantially to cell subtype heterogeneity [40,41,42] and manifest both advantages and disadvantages. On the one hand, this diversity mirrors the cellular environment observed in the native myocardium [43], thereby rendering 2D plate-format hiPSC-CM cultures invaluable tools for biomimetic inquiries. The broad spectrum of cardiac phenotypes and responses under diverse physiological and pathological conditions can be explored to identify disease mechanisms and drug reactions [44,45]. Studies utilising hiPSC-derived cultures containing diverse CM subtypes also provide insights into cardiac development, maturation kinetics, and subtype-specific disease manifestations [38,39]. Additionally, heterogeneous cultures may synergistically contribute to tissue regeneration and functional recovery following cardiac injury, which is beneficial in the context of regenerative medicine [28]. On the other hand, this cellular heterogeneity of hiPSC-CM cultures presents challenges, particularly when purity is paramount for specific applications like cell-specific drug interactions. Efforts have continued for the optimisation of CM differentiation protocols including the reprogramming and expanding of hiPSC colonies under feeder-free conditions, facilitated by specific culture media and substrates, and have contributed to isolating fully reprogrammed hiPSC colonies, thus enhancing the purity of the resulting CM populations [46]. Other efforts have considered factors such as cell seeding density and low concentrations of Wnt-agonists that have been shown to significantly impact the purity of hiPSC-CMs [47]. The pursuit of homogeneous hiPSC-CM cultures highlights the importance of refining co-culture conditions to replicate the intricate microenvironment of tissues or organs more effectively.

### 2.2. The Need for Co-Cultures and Subtype-Specific Cultures

While the necessity for purification and homogeneity is evident, recent insights suggest that the success of such endeavours may hinge upon co-culture conditions that are only beginning to emerge, thus introducing a layer of complexity similar to the heart [48]. Co-culture systems have demonstrated effectiveness in enhancing the differentiation of hiPSC-CMs. One approach involves co-culturing hiPSCs with cardiac fibroblasts (CF) and cardiac endothelial cells, which facilitates their differentiation into mature CMs [49]. Integrating these various cell types into co-culture systems holds promise for enhancing the tissue-like features and functionality of differentiated cardiac cells. However, the precise role of these additional cell types in co-culture systems remains poorly defined, and their integration presents practical challenges and complexities [45].

Much interest is placed in subtype-directed differentiation of hiPSCs into atrial and ventricular CMs, highlighting the importance of tailored differentiation strategies [50,51,52]. Coupling with advanced culture techniques such as microfluidic platforms or 3D tissue engineering systems offers enhanced control over CM subtype specification and maturation [45,50,53]. Therefore, the need to obtain CM cultures that are homogeneous and are subtype-specific is particularly important as there are electrophysiological variances among cardiac subtypes, such as atrial, ventricular, and nodal-like cells [54]. Each subtype displays distinctive action potential (AP) waveforms, indicative of their specialised functions within the heart’s electrical conduction system [54]. Much interest is placed in subtype-directed differentiation of hiPSCs into atrial and ventricular CMs, highlighting the importance of tailored differentiation strategies [50,51,52]. For example, CRISPR-Cas9 technology coupled with fluorescent cell sorting is an effective method for purifying atrial and ventricular populations from the same hiPSC line [55]. Several studies show the faithful recapitulation of disease-specific electrophysiological phenotypes in patient-derived cells [48,56]. As such, aberrant AP morphologies in specific CM subtypes may indicate underlying pathological conditions, such as arrhythmias, hypertrophic cardiomyopathy, or conduction defects [57,58]. Therefore, elucidating the molecular underpinnings governing CM subtype specification is essential for directing differentiation towards desired subtypes, thereby enhancing the fidelity of disease modelling and drug screening applications [59].

### 2.3. The Benefits of Gene-Editing Technology

Ultimately, the continued improvements in hiPSC-CM cultures, coupled with gene editing approaches like CRISPR-Cas9, present a powerful advancement for understanding specific gene mutations underlying cardiomyopathies. For example, such studies may help further our understanding of conditions such as hypertrophic cardiomyopathy, dilated cardiomyopathy, and arrhythmogenic cardiomyopathy, which are thought to be inherited and result from mutations in genes that encode proteins crucial for CM structure and function [60]. By introducing specific disease-associated mutations into healthy hiPSC-CMs, researchers can recapitulate and even discern spatiotemporal gene functioning of the disease phenotype in vitro. Studies have already shown the utility of introducing disease-associated mutations into healthy cells using CRISPR-Cas9 to investigate inherited cardiac channelopathies such as LQTS, Brugada syndrome, and short QT syndrome (SQTS) [61,62,63]. Much research is aimed at exploring the effects of these mutations on ion-channel function and on the electrophysiology profiles of CMs [63,64,65,66]. More recent advances show that hiPSC-derived cardiac disease models using precision gene editing and bioengineered 3D tissue models accurately recapitulated clinical phenotypes of inherited cardiac arrhythmias, providing robust validation of gene mutations and offering a powerful tool for dissecting pathophysiology in vitro [67]. Continued research efforts toward refining hiPSC-derived cultures, whether mixed, cell-specific, or even subtype-specific, will undoubtedly remain an ongoing endeavour in cardiovascular research.

## 3. In Vitro Cardiotoxicity

Cardiac safety is the main concern related to drug development during clinical trials and after release to the market. Many drugs have been withdrawn from the market due to cardiotoxicity, although a wider list of drugs is known to cause cardiac side effects. Antineoplastic drugs, central nervous system agents, genitourinary system agents, and anti-inflammatory, anti-infective, and cardiovascular agents are the most common to induce cardiotoxicity [68]. There are different mechanisms of drug-induced cardiotoxicity, including arrhythmias, disruption of mitochondrial function, apoptosis, altered growth factor signalling, or oxidative stress [68]. Correlation between animal models and human cardiac physiology is often complex due to the significant differences in heart rate (rats and mice have 6–10 times higher heart rate than humans) and the ability to increase heart rate and due to the dissimilarities in the main ion currents [69,70]. However, non-rodent models (rabbits, guinea pigs, dogs, and non-human primates) have a 90% chance of predicting QT interval changes in humans [71]. Given the current tendencies to reduce the use of animal models in science, in vitro cellular models are increasingly becoming essential tools for drug toxicity testing, including arrhythmias. The Comprehensive in vitro Proarrhythmia Assay (CiPA) collaboration, including regulatory agencies, industry, and academia from U.S. FDA, Europe, Canada, and Japan, created guidelines for cardiotoxic risk evaluation in drug candidates [72]. These include the in vitro assessment of drug effects on ion channels, computer modelling to predict risk based on drug effects on ion channels, effects in hiPSC-CMs, and phase 1 clinical testing in vivo [73,74,75,76].

The HOC platform is an automatic tool for large-scale drug testing, measuring electrophysiological and metabolic responses of CMs to different compounds [77]. HiPSC-CMs are the most promising model for cardiotoxicity tests as the primary cells are of limited availability and dedifferentiate rapidly while animal cell lines might provide false results because of differently functioning ion channels [78,79]. HiPSC-CMs can be used as an autologous or personalised system with specific genetic background. Additionally, patient-specific hiPSC-CMs can reflect the response from an individual patient in a personalised setting or represent the whole patient population, and these can be used for modelling various diseases of interest, including genetic mutations [80,81]. A typical HOC platform includes external electrical or mechanical stimuli, a calcium sensor, or a contraction detector. Validation of the HOC system can include testing drugs with well-known cardiac effects (e.g., isoproterenol) to serve as baseline controls [79]. On the other hand, the main concern about HOC platforms is the insufficient maturity level of CMs; therefore, the functional response might differ from the fully matured adult CMs [82].

### Cardiotoxic Effects and Their Detection

**Figure 5:**
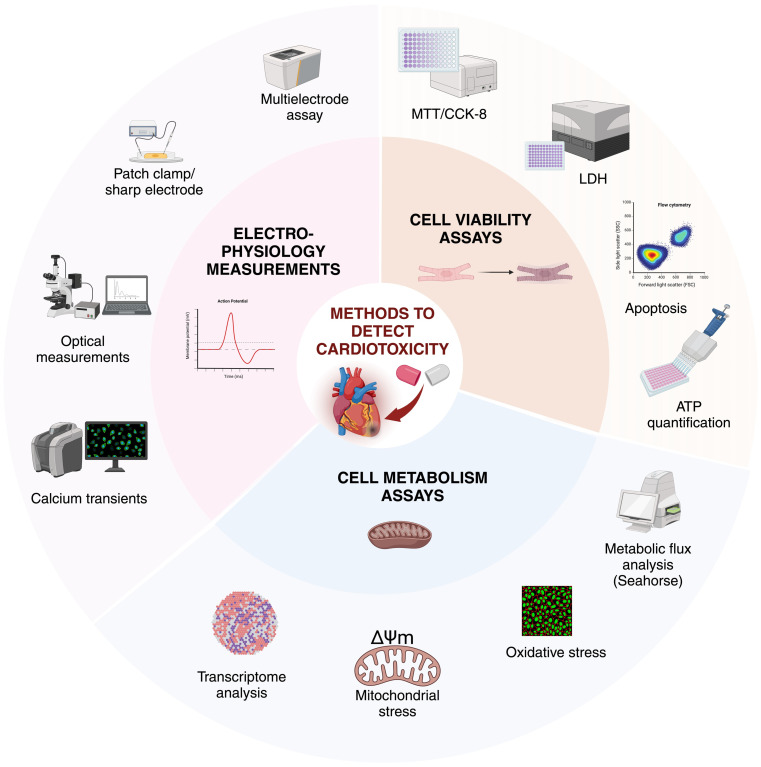
Methods to detect cardiotoxicity in CMs. In vitro, cardiotoxicity can be evaluated by a range of methods, including cell viability assays, cell metabolism assays, and electrophysiological readouts. Ion channel profiles, AP characteristics, and electrophysiological properties can be analysed by patch clamp, sharp electrode, or multielectrode measurements, in parallel with calcium transient kinetics and parameters. Cell viability can be monitored by ATP-release-based techniques, intracellular lactate dehydrogenase (LDH) activity, colorimetric assays such as MTT and CCK-8, and detected by a plate reader or by measuring apoptotic events. To quantify cell metabolism, the assessment of glycolytic and oxidative metabolism can be measured by Seahorse assays along with oxidative stress by measuring the toxic levels of ROS. Mitochondrial stress tests provide information on the resting respiration of the cells, by determining ATP production and oxygen consumption. Transcriptomic analysis and metabolomics focus on the underlying genes and their expression, the end products of these genes, and on the possible interactions in the metabolic pathways. Created with BioRender.com.

The main cardiotoxic effects include drug-induced arrhythmias or changes in cell metabolism or viability, which can lead to heart failure. There are many physiological processes that can be monitored for detecting cardiotoxicity in CMs, including electrophysiological assays, cell metabolism, viability, or oxidative stress (Figure 5). Drug-induced arrhythmias can be caused by many broadly used medications. One of the most serious conditions is QT interval prolongation, leading to torsade de pointes type ventricular tachyarrhythmias, although bradyarrhythmia, AF, tachycardia, or Brugada syndrome can also be caused [83]. It is known that blocking the potassium HERG channel leads to drug-induced prolongation of QT interval and is associated with sudden cardiac death [84].

Mitochondria account for almost half of human CM volume and are vitally necessary for energy production. During hiPSC-CM maturation, changes in the cell metabolism include the switch from glycolysis to fatty acid oxidation by oxidative phosphorylation, a decrease in glucose uptake, an increase in mitochondrial DNA content, an enhanced mitochondrial membrane potential, and a higher mitochondrial calcium level [53]. Cardiotoxic drugs might interfere with the mitochondrial respiratory chain, inhibit mitochondrial enzymes, induce loss of membrane potential, and increase oxidative stress [85]. If damaged mitochondria are not degraded, they start to accumulate, and it is associated with the pathogenesis of myocardial dysfunction [86]. For example, doxorubicin causes not only mitochondrial damage but the impairment of iron metabolism as well, resulting in the accumulation of reactive oxygen species (ROS) and leading to oxidative stress [87]. Mitochondrial damage can not only disrupt physiological processes in the myocardium but might also lead to cell death. CM death can be caused by several mechanisms, including autophagy, ferroptosis, apoptosis, pyroptosis, and necroptosis or DNA damage [88,89]. It has been shown that cardiac troponin I release in the medium correlates with cell viability data [90]. Consequently, the reduced viability of CMs can lead to structural damage to the myocardium.

The most important assays for detecting arrhythmias in vitro include multielectrode assays, patch-clamp and sharp electrode measurements, and optical evaluations of calcium transients or cell contractions providing analysis of multiple parameters such as the beat rate, field potential, or AP profiles (Table 1). Cell viability is generally measured by ATP production, troponin release, the metabolism of formazan-based dye (e.g., CCK-8, MTT, MTS), or by the intracellular lactate dehydrogenase activity [91]. These methods correlate with the number of living cells, while repeated measurements can be taken at different time points, making them suitable for long-term experiments. To evaluate drug effects on cell metabolism, cell respiration, oxidative stress, or mitochondrial membrane potential can be measured. Based on these readouts, a set of in vitro assays is available to estimate the potential damage in the myocardium caused by a substance (Figure 5).

Briefly, arrhythmias, altered metabolism, and reduced cell viability are major conditions representing drug cardiotoxicity that can be tested using in vitro cardiac models, including HOC. Furthermore, in vitro cardiac models may contribute to the elucidation of molecular mechanisms leading to drug-induced cardiotoxicity.

## 4. Scaffolds in Cardiac Applications

The field of cardiac tissue engineering has seen significant progress in recent years, with a focus on developing scaffolds that promote the maturation of cardiac cells. This section discusses the recent developments related to 2D and 3D scaffolds. The fabrication of 2D scaffolds involves the production of a stable and soft substrate with a smooth surface for cell growth and attachment. Several techniques are commonly used for fabricating 2D scaffolds. Soft lithography utilises elastomeric materials to create 2D patterns on a substrate, while micropatterning involves creating specific patterns or geometries using photolithography, microcontact printing, or microfluidics. Layer-by-layer assembly and various deposition and casting techniques are also employed for fabricating 2D scaffolds [107]. Electrospinning is a versatile technique that involves the creation of nanofibers using an electric field. Obtaining aligned fibers using rotating collectors is often the preferred approach [108]. Indeed, aligned fiber scaffolds offer advantages for tissue engineering applications, including cardiac tissue engineering, as they mimic the aligned structure of native tissues, such as in the myocardium, promoting directed cell growth and assisting the functional maturation of the cells. CM alignment can also be controlled using micropatterning techniques to favour cell adhesion along geometric pathways.

Two-dimensional CM cultures can simulate viable environments for cardiac cell maturation, which is a relevant and easy way of drug screening and toxicological testing with high throughput. These 2D cultures are often combined with microelectrode arrays (MEA) to measure the electrophysiology of the cardiac cells completed with optical imaging. Single-cell patch clamp and sharp electrode measurements in electrophysiology are also widely used on 2D cultures as they enable measuring the membrane potential, the upstroke velocity, and ion currents [107]. The disadvantage of these models is the limited complexity in the tissue construction without a physiological extracellular matrix (ECM), especially when using 2D monotypic models [109].

Alternatively, utilising 3D scaffolds offers several advantages over 2D scaffolds in cardiac tissue engineering. Most importantly, 3D scaffolds allow for the recreation of the complex architecture and microenvironment found in native tissues, facilitating appropriate cell–cell interactions, the formation of gap junctions, and tissue organisation. It is now well-established that other cell types such as CFs, macrophages, and endothelial cells are needed to favour the maturation of CMs and to recapitulate specific functions of the native cardiac tissue [110]. Moreover, 3D-vascularised scaffolds improve nutrient and oxygen diffusion, promote cellular organisation and polarisation, and offer a physiologically relevant platform for disease modelling. In addition, 3D scaffolds allow the use of spatial, electrical, and mechanical cues, which are crucial for cardiac tissue development and maturation. The physical properties of 3D scaffolds such as stiffness, topography, and porosity can be precisely controlled to mimic the native mechanical environment. Different construction methods include 3D aggregation, application of hydrogels, and micromold design to obtain engineered cardiac tissues (ECT) around anchor pillars or 3D bioprinting [107,111]. It is important to underline that 3D cardiac tissues can be fabricated with or without scaffolds [112,113]. Layer-by-layer cardiac tissues, cardiac spheroids, and organoids can be obtained without scaffolds while the use of hydrogels, decellularised ECM, and microfabricated 3D scaffolds are alternative strategies to obtain 3D cardiac tissues using 3D scaffolds with different geometries. For the fabrication of cardiac patches, the latter approach is usually preferred [114].

In these applications, a crucial issue is the sufficient supply of oxygen and nutrients without a proper vascularisation of the native 3D tissue. The use of microfluidic systems, sacrificial materials, or 3D printing of vascular channels has been explored to overcome this issue [115]. While 3D scaffolds provide a more biomimetic and functionally relevant platform, shifting toward HOC systems presents significant challenges. These include the complexity of the cardiac microenvironment, integration of multiple cell types, and achieving functional maturity of CMs.

## 5. Heart-on-a-Chip Platforms

**Figure 6:**
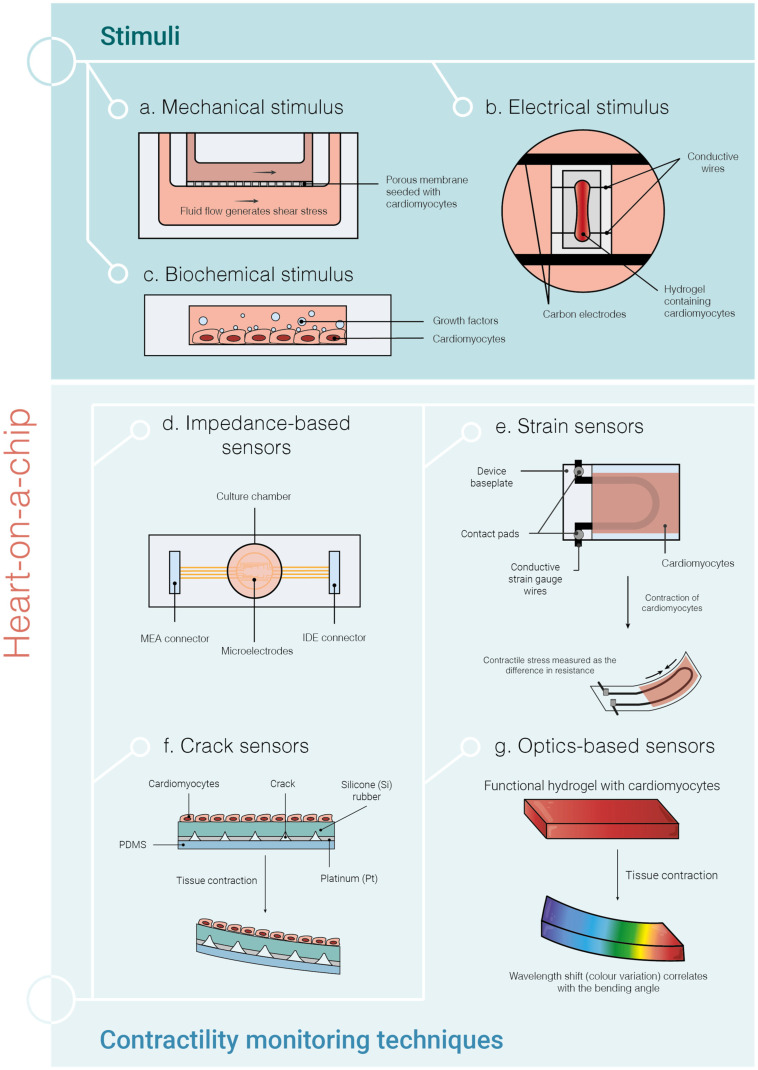
Current strategies of heart-on-a-chip (HOC) systems using hiPSC-CMs. The microfluidic HOC systems serve as controlled environments to mimic as much as possible the physiological conditions that can be found in the human native heart. The most important stimuli methods (**a**–**c**) and contractility monitoring techniques are shown (**d**–**g**). Mechanical stimulation (**a**) provides mechanical cues to the cells, which may be shear stress (by exposing the cells to fluid flow) and/or stretch forces. Biochemical stimulation (**b**) consists of applying growth factors to the cells and electrical stimulation (**c**) can be achieved by applying small electrical pulses to the CMs. Impedance-based sensors (**d**) measure cardiac contraction by reading the impedance changes upon contraction of the CMs. Strain sensors (**e**) rely on the contraction of CMs to change the curvature of the device baseplate and measure the difference in resistance, which is then converted by a mathematical formula to the stress generated by the tissue. In crack sensors (**f**), the cracks in the sensor open as the tissue contracts, increasing the resistance. The contraction of the CMs causes the deformation of the hydrogel substrate leading to a wavelength shift (colour variation), which can be measured with optical sensors (**g**). MEA—multielectrode array; IDE—interdigitated electrodes.

A HOC is a microfluidic device capable of mimicking the complex physiological conditions of the native heart tissue and delivering electrical, mechanical, and/or biochemical cues under a controlled environment [116]. Mechanical (stretch forces and fluidic shear stress), biochemical (e.g., vascular endothelial growth factor and fibroblast growth factor), and electrical stimuli have an influence on cell differentiation, alignment, and physiological behaviour (Figure 6) [117]. Cell culture medium perfusion, i.e., fluidic shear stress, is widely applied in HOC devices with the aim of evaluating drug responses. For instance, Christoffersson et al. attached cardiac spheroids formed by hiPSC-CMs to a microfluidic device channel coated with laminin to assess drug toxicity [118]. The drug effect was quantified by observing the cardiac cell outgrowth from 3D spheroids under perfusion and it was validated using six compounds with well-established effects on cardiac cells. Despite the sensitivity of the assay, it is worth noting that the CMs used in this study had an immature phenotype and the model is not completely representative of a 3D model. However, the easy handling of the system allied to its cost-effectiveness is advantageous, especially if it is used as a complementary tool to the existing assays. In another study, the tissue was stimulated with fluid flow, and the platform contained a vascular endothelial layer to assess the significance of microvessels in drug toxicity experiments [119]. The dynamic conditions showed reduced dextran permeation compared to the static conditions; the viability of both human umbilical vein endothelial cells (HUVECs) and hiPSC-CMs was slightly improved under fluid flow, and HUVECs aligned according to the flow direction. Hence, fluidic shear stress-induced cell alignment and mimicked in vivo forces.

Electrical stimulation in hiPSC-CMs directs the cell differentiation process towards a more mature phenotype [120]. Therefore, this type of stimulation has also been incorporated into HOC platforms. For example, electrical conditioning was used to stimulate the maturation of atrial and ventricular hiPSC-CMs, which were embedded in a hydrogel together with CFs, cultured inside the Biowire II platform [121]. This stimulus improved the sarcomeric organisation in both atrial and ventricular tissues and promoted the expression of maturation genes associated with contraction in the ventricular construct, calcium handling, electrical properties, and lipid metabolism.

In addition to providing different types of stimuli to CMs, HOC systems enable the integration of sensors for continuous monitoring of tissue contractile functions [122]. This function can be measured using impedance-based sensors [123], strain [124,125], and crack sensors [126]. Furthermore, tissue contraction can also be assessed optically by changing the curvature of the cantilever [127] and by capturing images of the tissue [82] or the hydrogel colour change [128,129,130] (Figure 6).

Importantly, the incorporation of iPSCs derived from patients with specific diseases into HOC systems is extremely valuable either to evaluate drug toxicity in a targeted patient or to develop new and tailored treatments [116]. Despite the efforts in developing HOC for different applications (e.g., research on the mechanism of heart diseases, drug development for CVDs, development of medical treatments, and drug toxicity testing) and their promising results, these devices have several limitations. They fail to replicate the complexity of the native heart, only incorporating two or three cell types. Indeed, the incorporation of macrophages, neurons, epicardial cells, and endocardial cells is yet to be successfully accomplished. Moreover, the dilution of secreted biomolecules in the circulating fluid hinders their detection [131]. Currently, hiPSC-CMs remain immature compared to human adult CMs, which hampers their use in pharmacological and toxicological screening. New methodologies for hiPSC-CM maturation must be incorporated in HOCs, e.g., integrating conductive spheroids or scaffolds (hydrogels and membranes) into the platforms. These systems must evolve toward the use of low-cost and disposable materials to be attractive to pharmaceutical companies and research institutions. In the case of more expensive components, such as sensors or motors, these should be reusable and easy to sterilise. The development of multi-organ-on-a-chip and human-on-a-chip seems to be a logical direction to pursue, not only for highly predictable drug toxicology studies but also to study the interactions between different organs. However, the complexity of such platforms together with the need for a universal cell culture medium suitable for all organs present significant challenges. Innovative approaches and solutions are still needed to overcome these obstacles and accelerate the adoption of HOC systems in preclinical testing.

## 6. In Silico Models

The use of mathematical and computational models plays an important role in comprehending complicated biological processes. These models promote the identification of basic behaviours, laying the groundwork for an extensive characterisation and a more profound understanding of the underlying natural processes. The work from Hodgkin and Huxley is an early example of a mathematical model intended to imitate electrical signal generation [132]. Ever since, the application of in silico modelling has become foundational in cardiac electrophysiology and has shown to be a reliable method that can successfully connect diverse datasets and precisely define cellular characteristics that are responsible for the observed variability in experimental outcomes.

One of the most extensively studied functionalities of CMs is the AP. The collection of AP data is typically carried out using sharp microelectrode or patch clamp techniques. Calcium transient can also be measured through fluorescent imaging and processed to time series [133]. These data are then characterised through computational modelling to depict fundamental behaviours, as it is illustrated by Akwaboah et al. with their implementation of genetic algorithms to accurately reproduce experimental data to validate AP morphology and various ion channel blocking mechanisms [134]. Of great importance, contractile properties of hiPSC-CMs could also be monitored and analysed [135]. Additionally, data on transcription factors have the potential to model the differentiation state of CMs, as their expression changes upon differentiation [136,137].

Usually, primary data from hiPSC-CM cultures undergo processing before model fitting, with derived biomarkers forming the basis for model development. An effective manner to extract biomarkers is through specialised software for automated quantitative analysis of specific CM parameters, which offers a user-friendly approach to analyse data. The ‘Cardio PyMEA’ software (https://github.com/csdunhamUC/cardio_pymea?tab=GPL-3.0-1-ov-file, accessed on 30 July 2024) allows users to analyse MEA data from CMs, from beat detection to biomarker analysis such as beat amplitude and interval [138]. SarcGraph v0.2.1 is an open-source Python-based software that extracts data from CM culture videos of fluorescently labelled contractions [135], which can build upon previous research works [139,140]. These solutions automatically detect and track the sarcomere and z-disc allowing for spatio-temporal post-processing and visualisation. Sarcomere analysis is performed through scanning gradient Fourier transform to the organisation and alignment of sarcomeres [141]. Other contraction tracking options are also available, such as plug-in tools to existing software [142] testing out state-of-the-art methods [143] or focusing on the micro level [144]. Alternatively, images or videos have been directly analysed through deep learning (DL) and machine learning (ML) to generate models for detecting abnormal cultures [145,146]. ML—a subset of artificial intelligence (AI)—enables systems to autonomously learn and enhance from data, utilising various algorithms for tasks such as classification (e.g., decision tree), clustering (e.g., k-nearest neighbours), and prediction (e.g., linear regression). While DL—a subfield of ML—employs layered artificial neural networks to do analogous tasks. The primary limitation of ML methods is their requirement for substantial data, with DL being particularly data intensive. In general, the main drawback of mathematical modelling is that it heavily depends on the quantity and quality of real-world observations.

An important application of hiPSC-CM cultures is the evaluation of candidate drugs in a human model [147]. AP, contraction, and calcium transient are used to monitor drug effects on CM cultures. AP data are particularly valuable for generating computational models of cell cultures, where the effects of drugs can predict the behaviour of the CMs [148,149]. This was illustrated by Jæger et al., who sought to predict the effects of drugs by modelling the AP of cells both with and without the SQT1 mutation associated with SQTS [150,151]. Furthermore, a software tool has been implemented to account for uncertainties in dose–response measurements of drugs in cultures, translating them into prediction models and providing a probability distribution for the model’s outcome [152]. Functional analysis of CMs has also been conducted through video-derived data, such as contraction speed, BPM, and calcium transient. ML models have been developed to assess and classify the effects of drugs in screening assays through the characterisation of AP [143,153,154]. In 2021, Grafton et al. introduced a DL model specifically designed for the rapid identification of cardiotoxicity patterns from images. This model was refined through high-content image analysis of treated hiPSC-CMs across over 1200 compounds [141]. The integration of these models into automatic high-throughput screening processes enables the possibility of conducting large drug screenings efficiently.

HiPSC-CMs exhibit significant versatility in altering their functionality, induced through mutations or specific culture conditions [155,156]. Therefore, developing models capable of rapidly and accurately identifying abnormal changes is essential. Hwang et al. proposed a DL model coupled with an analytical algorithm using images that tracked calcium transient [157]. The model aims to distinguish normal and abnormal behaviour of hiPSC-CMs. These models led to the development of a range of tools commonly applied in hiPSC-CM research, including abnormal AP detectors or tools for identifying LQTS. Moreover, brightfield microscope images and videos, quantifying contraction properties, have been used to train DL models aimed to characterise the hiPSC-CM cultures before commencing an experiment [145,146]. In addition, the iMATURE v1.1.2.3 software is a tool that allows the users to manually select the age of hiPSC-CM post-differentiation and receive predictive readouts of AP morphology and ion channel dynamics over their specified age range [158]. This model is built upon the work of Kernik et al. from 2019, where they collected experimental data from different laboratories to model experimental variability and describe subcellular mechanisms [159].

Characterising cell lines is pivotal in cellular research, and models play a significant role in this regard. Notably, there is a maturity level discrepancy between hiPSC-CMs and primary CMs [137,160], e.g., hiPSC-CMs may lack the completion of critical perinatal processes [161]. Additionally, Paci et al. demonstrated in 2015 that models for hiPSC-CMs and CMs are not interchangeable [162]. Subsequently, it has been recognised that models should be tailored for specific cell lines to enhance predictions of baseline behaviours [149]. For instance, there are differences in gene expression profiles during the differentiation stages [137], in fact, regression models can define CM culture age with gene expression biomarkers data as the input [136]. Therefore, understanding the intricacies of cellular growth, differentiation, and proliferation is also crucial in research, and the models play a supportive role in deciphering the regulatory processes within these crucial aspects of wet lab work. Previous works serve as comprehensive resources for theoretical frameworks to predict differentiation fate or cellular growth [163,164]. Although the adjustment of culture media was a promising method for enhancing hiPSC-CM maturation, no specific modelling approaches have been identified, but there are studies on modelling techniques applied to optimise cell culture formulation [165] and scaffold construction [166].

Typically, biomarkers are derived from raw data to model hiPSC-CM behaviour, and the ability to generalise insights from computational models relies on acquiring substantial amounts of high-quality data. It is important to recognise, though, that gathering this kind of data is hindered significantly by the cost of experimental observations. Today, when modelling approaches like DL are popular and promising, we must keep in mind that their efficacy depends on the availability of significant and reliable data. Although data scarcity in hiPSC-CMs is still an issue, using mathematical and computer models is key to understanding complex biological processes because they make it easier to identify basic behaviours, which lays the groundwork for more detailed characterisation and a better understanding of the underlying events. Thus, the synergy between cellular and computational models offers the potential to streamline processes, reduce the need for animal models, cut down costs, and enhance the reproducibility of scientific experiments.

Besides modelling using hiPSC-CMs, in silico models have also been used to directly simulate primary CMs, thus allowing mathematical modelling of cell function [167] and also dysfunction in heart diseases such as ischaemia or heart failure [168,169]. Regardless of the origin, cellular models carry individual variabilities to some extent, which might be pivotal in predicting diseases or treatments; however, hiPSC-CMs are considered as models with reproducible genetic backgrounds and thus less variability than primary cells. On the other hand for modelling the human population, there is a need for greater genetic diversity among the hiPSC models [170]. This might increase the resources needed and reduce the practical benefits of the in vitro assays. Recently, to overcome the issues of individual differences, ML models were trained on large datasets of CMs to detect individual variability and predict susceptibility to arrhythmias [171]. The integration of in silico models with experimental data leads to the development of digital tools that can be implemented in functional cellular studies and to study the impact of a drug on cardiac tissue. The experimental data serve as an input for a previously trained computational model, which can swiftly identify functional changes without the need for complex experiments and, in parallel, reduce the use of laboratory animals.

## 7. Disease Modelling

### 7.1. Atrial Fibrillation

AF is the most prevalent cardiac rhythm disorder, affecting 59 million people globally in 2019 [172]. The incidence and prevalence of AF are rising significantly, considering this disorder as a global epidemic [173]. Despite significant advances in detection and management, AF continues to have a major impact on the morbidity and mortality of millions of patients [174], partly due to unresolved knowledge gaps in AF pathophysiology and therapeutic strategies, including rate/rhythm control and stroke prevention [175]. Additionally, the response in an individual patient to antiarrhythmic drugs is highly variable, in part due to the inability to target the underlying genetic mechanisms of AF. Indeed, current pharmacological options remain inefficient, with substantial adverse side effects, including drug-induced proarrhythmia, and both cardiac and non-cardiac toxicity [175,176]. The limited efficacy of current pharmacological treatment options and the need for a more complete understanding of the pathophysiology of AF require more advanced human models, allowing the development of a powerful personalised treatment for AF. Due to the high interspecies variabilities, in vitro models with human CMs represent a promising human-based tool to study mechanisms and processes involved in AF. While it is possible to isolate adult heart cells from patients after heart surgery, several drawbacks exist, including severe source limitation, the non-proliferative feature of human CMs in culture, and consequently a precipitous functional decline in vitro, severely limiting their application for larger-scale drug screening and disease modelling [38]. In contrast, hiPSCs can be differentiated into atrial CMs (hiPSC-aCMs), providing a nearly unlimited source of cells that can be used to model disease at a single cell level, reproducing the human cardiac electrical phenotype in health and disease [177]. Since gold standard differentiation protocols to obtain hiPSC-CMs yield a mixture of different CM subtypes, such as ventricular-, atrial-, and nodal-like cells [36,43,178], significant efforts have been made to establish differentiation protocols and/or selection strategies to generate and purify a population of chamber-specific CMs [52,179,180]. The potential of generating such chamber-specific cell-type models significantly improved the research for disease modelling and drug testing applications. Atrial-like CMs are particularly suitable for modelling atrial arrhythmias, such as AF. Nevertheless, to generate relevant models for studying the mechanisms involved in AF, the patterns of AP propagation occurring in the human heart during both sinus rhythm and arrhythmia should be properly recapitulated in vitro. Moreover, while it is pivotal to closely mimic the anatomical features of the atria and their function to investigate specific disease pathways or arrhythmia mechanisms, the model should be applicable in a standardised, systemic, and controllable manner [181]. One limitation of using hiPSC-aCMs as a well-established model for AF concerns the method to generate the cells, which, most of the time, is laborious, time-consuming, and costly and, therefore, hard to scale up. To improve the stability and reproducibility of the model, studies focused on the optimisation of the protocol to obtain hiPSC-aCMs in vitro [52,182,183]. Recently, Thorpe et al. investigated the timing of retinoic acid addition during CM differentiation, with the aim of developing a robust and scalable protocol to produce large amounts of atrial-specific CMs [184]. The protocol was successfully applied to six different hiPSC lines, without the need for line-specific optimisation. The authors could elicit arrhythmic activity in response to burst pacing, highlighting the potential of the model as an antiarrhythmic drug screening platform, and investigating human atrial arrhythmias and myopathies. Similarly, Schulz and colleagues highlighted the variability in the expression of atrium-selective currents, suspecting methodological issues in producing hiPSC-aCMs [185]. These results showed that only 1 μM retinoic acid induced enough ultra-rapid delayed rectifier potassium current (I_Kur_) to fully reproduce human atrial AP shape.

Besides the improvement and refinement in protocols to generate hiPSC-aCMs, these cells still phenotypically resemble foetal atrial CMs. This represents another limitation of the model, and numerous maturation strategies have been implemented [1]. Among several methods, co-cultures have been recently implemented in the context of AF models [177]. Because of pivotal cellular interactions, co-culture with CFs has been used to improve hiPSC-CMs maturity [49]. Nevertheless, atrial CFs substantially differ from ventricular CFs, as they produce chamber-specific ECM protein isoforms, respond differently to key growth factors, and have distinct proliferation rates [186]. For these reasons, the effects of chamber-specific fibroblasts on hiPSC-aCM maturation and function remain unclear. Recently, Brown et al. reported that primary adult atrial fibroblasts induced higher functional maturation in hiPSC-aCMs [177]. Together with a soft-lithographic process to generate patterned co-culture of hiPSC-aCMs and ACFs, the work showed a significant improvement in hiPSC-aCM features (i.e., electrophysiology and metabolism) as compared to conventional and randomly distributed co-cultures. Moreover, this method showed a higher sensitivity for drug screening and disease modelling of familial AF [177].

The inclusion of other cell types has also been used in 3D models to generate engineered heart tissues (EHTs). The cellular complexity of these models can produce more accurate results, incorporating cells that can interact and communicate in a 3D environment, which is more representative of the physiological state of the cardiac tissue [187]. Compared to monolayer cultures, the EHT format highlighted atrial versus ventricular differences in vitro, demonstrating the strength of this method to generate atrial-like muscle structures in terms of gene expression, contractile force, contraction kinetics, and AP [188].

Finally, a growing understanding of the genetics underlying AF has enabled new treatment possibilities, allowing a deeper understanding of the disease pathogenesis [189]. Moreover, due to the increased percentage of lone AF cases, efforts have been made to study the underlying genetic contribution to AF, with the aim of facilitating early identification of people at high risk of developing this disease later in their lives [190]. Among several genome editing techniques, the CRISPR-Cas9 system has mainly been used for somatic genome editing of the heart to study disease phenotype and therapeutic interventions [191]. This system allowed the development of isogenic hiPSC disease lines of AF, enabling the study of patient-specific disease mechanisms and setting the stage for a pharmaco-genomic screen. Indeed, the use of unrelated controls and the failure to correct AF-causing genes (to generate an isogenic hiPSCs line) may provide only limited insights into the underlying pathophysiological mechanisms of familial AF [192]. Recent studies investigated underlying genetic mutations linked to AF, including isogenic controls and, hiPSCs from unaffected family members, not harbouring the mutation. For example, in the study of Hong et al. patient-specific AF hiPSC-aCMs exhibited remarkable in vitro phenotypes of AF-linked *SCN5A* mutations [193]. The use of a hiPSC line not harbouring the *SCN5A* mutation and the gene corrected-isogenic control line allowed to examine important insight into genotype-phenotype correlation, confirming the E428 variant as the cause of the AF. Similarly, Sumer et al. used hiPSCs to precisely correct heterozygous *SHOX2* mutations associated with AF, contributing to elucidating the function of *SHOX2* in the genetic network of atrial and nodal CMs and its contribution to the development and progression of AF [194]. A recent review article discussed the use of hiPSC-CMs as a model to study the role of small-conductance calcium-activated potassium channel (SK) variants associated with AF. The authors highlighted the advantages of using the hiPSC-CMs model to investigate single nucleotide polymorphisms (SNPs) associated with AF, addressing limitations and best practices for rigorous hiPSC studies, including the need to minimise off-target mutagenesis and the need to include isogenic controls to better elucidate the impact of a single variant [195]. Consequently, improving gene-editing studies will pave the way to model pre-clinical testing of antiarrhythmic drugs for a more personalised approach to AF therapy.

### 7.2. Hypertrophy

Cardiac hypertrophy is an adaptive response to pressure or overload, resulting in altered gene expression, metabolism, and cell morphology. Hypertrophy can be physiological or pathological. Physiological hypertrophy is characterised by a 10–20% increase in CM length and width, which is caused by postnatal growth, pregnancy, or high endurance exercise, and it is not related to dysfunction [196]. Whereas, pathological cardiac hypertrophy is caused by myocardial injury, stress induced by hypertension, or neurohumoral regulation leading to fibrotic remodelling, cardiac dysfunction, heart failure, and even death [197]. The main regulators of hypertrophic response are calcineurin/nuclear factor of activated T cells (NFAT), mitogen-activated protein kinase ERK, small guanosine triphosphate (GTP)-binding proteins (Ras, Rho), protein kinase C (PKC), and others [198]. To better understand molecular mechanisms and possible treatment targets of hypertrophy, in vitro models are widely used.

In vitro, hypertrophic phenotypes can be characterised by morphological changes, such as increased cell and nucleus size, upregulated expression of classical hypertrophy markers (foetal genes, ANP, BNP, and ACTA1, β-MHC), cell cycle arrest, and metabolic switch towards the foetal-type under the increased reliance on glucose [199,200,201,202,203,204,205]. Increased glucose consumption is one of the features of hypertrophic response and it can be measured by increased lactate concentration [203]. Multiomic approaches may give a deeper understanding of mechanisms of hypertrophy. For instance, transcriptomic analysis has shown enhanced expression of genes related to muscle contraction, myofibril assembly, and maturity-related structural elements of the cytoskeleton as well as altered expression of genes encoding calcium handling proteins in hypertrophic CMs [203,206]. Elevated expression of COL12A1 and THBS1 genes was also detected in a hiPSC-CM model of endothelin-1-induced hypertrophy, implying a relation to fibrosis and cardiac remodelling [203]. Notably, proteomic analysis revealed that the pathways of cardiac hypertrophy signalling, actin cytoskeleton signalling, and the superpathway of inositol phosphate compounds and PPARα/RXRα activation were changed significantly in hypertrophic CMs [207].

For modelling hypertrophy in vitro, neurohormonal or mechanical approaches are used. Endothelin-1, phenylephrine, isoproterenol, norepinephrine, and angiotensin II are classically used drugs to develop hypertrophy in CM cultures. Increased pressure or overload is introduced using physical stimuli, such as cyclic mechanical stretch [206]. Moderate mechanical stretch is also an option for CM maturation, while high afterload can cause hypertrophic changes in hiPSC-CMs [208]. Activation of glucose metabolism by testosterone or prolonged cultivation under high glucose conditions combined with endothelin-1 and cortisol stimulation can be utilised to induce diabetic hypertrophy in hiPSC-CMs associated with the accumulation and peroxidation of lipids, altered calcium handling, and loss of sarcomere integrity [209,210,211].

Novel approaches including patient-derived materials, such as endothelial cell-derived microvesicles (EMVs) from obese/hypertensive patients can be used to induce hypertrophy with increased levels of hypertrophic markers, such as cTnT, α-actinin and NF-kB, and fibrosis marker TGF-β in hiPSC-CMs [212]. HiPSCs from patients with genetic mutations (i.e., c.478_480del and p.Δ160E) in TNNT2 or hypertrophy-related diastolic dysfunction are also accessible for testing drug responses and for novel drug developments due to their elevated diastolic intracellular calcium levels and altered calcium handling [212,213].

Cardiac organoids serve as promising models to study hypertrophic changes in a mixed 3D culture, even if hiPSC-CM-based models are still lacking. Hypertrophic effects of bisphenol A (BPA) and bisphenol AF (BPAF) were studied in cardiac organoids, composed of hiPSC-CMs, human primary cardiac fibroblasts, and human endothelial cells. Results showed that contraction and calcium transient amplitudes were decreased and the level of proBNP increased [214]. Therefore, data about fibrosis in hypertrophy are still missing in both 2D and 3D models. Taken together, the good response of hiPSC-CMs to hypertrophy-inducing chemical or physical approaches makes them a valuable tool for drug development in hypertrophy-related cardiac diseases. Furthermore, a successful hiPSC-CM maturation strategy for adult phenotypes might provide a more suitable basis for hypertrophy studies.

### 7.3. Channelopathies

The shape of the APs in CMs is defined by the balance of inward and outward currents flowing through sodium, potassium, and calcium channels [215]. The disruption of functionality in any of these channels, or channelopathies, may lead to arrhythmias or other pathological states [216]. The most common cause of channelopathies is mutations in genes encoding ion channels [216]. The mutation may cause subtle and multiple alterations in ion channel activity, including modulated channel conductance, shifted voltage dependence of activation, or altered channel gating kinetics [215]. This is where the hiPSC technology has provided significant progress by revealing the mechanisms of channelopathies. Electrophysiological investigation in patient-derived hiPSC-CMs enables the determination of patient-specific biophysical consequences of a channel mutation [215,217]. Moreover, it provides the possibility of finding personalised and safe treatment strategies [33].

LQTS is considered one of the most common types of cardiac channelopathies [216]. The delayed repolarisation in LQTS can be caused by different reasons. For instance, LQTS subtype 1 is caused by the mutation in the KCNQ1 channel [216,218], while LQTS subtype 2 is caused by the mutations in the KCNH2 channel, resulting in reduced potassium currents through the respective channel [219]. Brugada syndrome is a rare inherited cardiac arrhythmia, associated with mutations in a number of genes [220,221]. Gene mutations of sodium voltage-gated channel alpha subunit 5 (SCN5A, encoding the ion channel Na_v_1.5) are responsible for 30% of Brugada syndrome cases. SCN5A mutations result in a constant inward sodium current during the plateau phase of the AP and a prolonged QT interval [222]. Whereas, another mutation associated with Brugada syndrome in plakophilin-2 results in deficits of sodium current [223].

HiPSC-CMs can be implemented for modelling a number of other cardiac channelopathies, including SQTS [224], Timothy syndrome [225], catecholamine-induced polymorphic ventricular tachycardia [226], or channelopathy of small- and intermediate-conductance calcium-activated potassium channels [195,227]. For a more detailed review of the correlation of calcium handling defects and channelopathies, see our previous review by Kistamás et al. [228]. The phenotypic outcomes during the modelling of the cardiac disease may be influenced not just by presumed causative mutations, but the differences in the genetic background as well. The precise introduction of desired mutations in a known hiPSC line can be performed by CRISPR-Cas9 techniques. This approach was used recently to model LQTS and SQTS phenotypes by introducing a specific mutation of KCNH2 in isogenic hiPSC-derived cardiac tissues [67].

One of the main shortcomings in the use of hiPSC-CMs for modelling channelopathies is their immature phenotype. The hiPSC-CMs demonstrate spontaneous activity with a depolarised membrane, low maximal upstroke velocity, and highly variable action potential duration [229]. Also, the expression of the inward rectifier potassium current in hiPSC-CMs is almost negligible in the foetal phenotype [229,230]; however, there are promising studies showing an increased density of this current upon metabolic maturation medium [231]. Therefore, the shape of the AP and the profile of the individual membrane currents contributing to it substantially differ from those observed in adult human CMs. While there were some attempts to facilitate the expression of certain ion channels by adenoviral techniques [232] or enhance them with dynamic clamp [233], the new techniques for enhanced hiPSC-CM maturation will open new possibilities for the investigation of the mechanisms behind channelopathies and develop novel treatment strategies.

### 7.4. Ischaemia/Hypoxia

A high number of CVD patients experience chronic or acute ischaemia. Chronic ischaemia is caused mostly by coronary artery disease, leading to chronic heart failure and ischaemic cardiomyopathy [234]. Acute myocardial ischaemia is one of the causes of sudden cardiac death, caused by the obstruction of coronary vessels, leading to the lack of oxygen and nutrients and the accumulation of waste products in the myocardium. Ischaemia disrupts cellular metabolism and ion currents leading to lethal arrhythmias [235]. Under ischaemic conditions, metabolism in CMs switches to anaerobic, and the generation of lactic acid is followed by the development of acidosis, which in turn leads to the reduction in ATP availability. If ischaemia persists, bradykinin, histamine, and ROS are released, disrupting CM membranes and leading to changes in electrophysiology [236].

Cardiac ischemia models in hiPSC-CMs can be induced by the application of low oxygen concentration (≤2%) in the absence of glucose and serum. The main responses to ischaemic conditions consist of reduced cell viability, contractility (reduced beating frequency, increased depolarisation time, and field potential propagation), and reduced sarcomere coverage and nuclear size, representing myocardium damage and arrhythmias in vivo [237,238,239]. Exposure to 2% oxygen concentration reduces the viability of hiPSC-CMs, which can be easily indicated by staining of the nuclei with propidium iodide [237]. Modelling acute ischemia (0–1% oxygen and glucose-free) can be combined with reperfusion (recovery in normoxia conditions of 19–20% oxygen, serum-free/glucose-free, or full media), together with hyperkalaemia and acidosis [240,241]. It causes cell apoptosis at the reoxygenation phase corresponding to the injury in the myocardium of patients when circulation is restored following a heart attack or cardiopulmonary bypass surgery [241,242]. The advantage of the application of hiPSC-CMs over the proliferative CM cell lines (e.g., AC16, HL-1, and H9C2) was shown in simulated ischaemia–reperfusion and hypertrophic settings [243]. Ischaemia simulation in hiPSC-CM cultures using 1% oxygen resulted in arrhythmias, which were determined by the analysis of the parameters of calcium transients, such as beat rate, diastolic calcium levels and calcium transient amplitude, irregular phases, double or multiple peaks, prolonged rise, plateau abnormality, and low or high peaks [239]. Whereas, 0% hypoxia-induced electrophysiological changes, such as decreased beating frequency and field potential amplitude, could be reverted during the reoxygenation phase. The reversion of hypoxia-induced morphological changes, such as disruption of the distinct sarcomere structure or a decrease in the nucleus area was not determined [244]. Metabolic purification can also be used for the induction of ischaemia as it increases susceptibility to hypoxia by inhibiting mitochondrial respiration, which can lead to cell death [245,246]. Metabolic purification was shown to reduce sarcoendoplasmic reticulum calcium-ATPase (SERCA) expression, as cells were depolarised and had lower mitochondrial membrane potential [245]. In summary, by reducing the concentration of oxygen in cell cultures and thus recapitulating the microenvironment of ischaemic diseases, it is possible to reproduce acute or chronic changes in cardiac models using hiPSC-CM models, such as alterations in cell viability, electrophysiology, calcium handling, or to study reperfusion-induced injuries after the reversal from hypoxic to physiological oxygen levels in the culture environment.

## 8. Conclusions and Future Perspectives

We conclude that with the advent of hiPSC-CM technology and tissue engineering models, configurations resembling native tissues may be the optimal direction in drug screening and disease modelling. However, significant limitations persist in the field that first need to be addressed to make it a reliable tool. The most prominent drawback of these cells and models is their insufficient maturity status. Nonetheless, numerous studies demonstrated that these human cells are superior to most animal models, and they can offer several advantages over human primary cells. Importantly, employing these cells is expected to enhance adherence to the principles of 3Rs (Replacement, Reduction, and Refinement) in the use of animals in research. The continuously growing data pool collected from hiPSC-CMs serves as a ground for extensive in silico modelling, which can identify particular patterns and biomarkers associated with CVDs and can also predict drug-induced alterations. On top of that, computational simulations and models might promote diagnostic purposes, either by earlier diagnosis or by easier and more accurate follow-up of patients.

Besides the human origin, the pursuit to establish a pure cell culture has two sides. First, a pure culture having only the desired cell type is a major goal in many studies, but there are studies showing that without co-culturing with other cell types, genuine maturation cannot be achieved. Ongoing clinical trials show encouraging results with pure cultures as a great option for treating heart muscle injuries during myocardial infarction in vivo. In vitro, however, adding supporting cell types to have a mixed population appears to be beneficial for CM maturity. Connecting several organ-on-a-chip platforms to establish a multi-organ-on-a-chip, also known as a human-on-a-chip platform, could allow us to assess not just hidden cardiotoxicity, but to identify effects of complex toxic environments, such as air pollution, on different artificial mini organs. Although hiPSC-CM sources are theoretically unlimited, there are still many ongoing attempts at their large-scale expansion for clinical and in vitro applications. In terms of financial aspects, it is key to establish ready-to-use robust, reproducible, and cost-effective protocols in industrial and medical quality and quantity production.

The demand is high for human disease models as in the case of atrial fibrillation. The key limitations are that there are no such gold standard protocols that would be ideal for all hiPSC lines, the low maturity status, and the imperfections in gene editing. The main limitation of personalised medicine solutions is the limited understanding of the underlying patient-specific mutation linked to the disease. Even if major advancements have been achieved with genome-editing techniques (e.g., CRISPR-Cas9), it is still not straightforward to investigate the phenotype associated with specific mutations. Notably, there is a pivotal need for isogenic controls. However, progress has been made and several works showed that including isogenic and healthy controls (not carrying the mutation) will be extremely relevant for personalised medicine. Next-generation sequencing (NGS) technologies have enabled fast and affordable gene-based diagnostics, but an important challenge persists due to the limited comprehension of genetic variants in detected disease-associated genes. Enhancing our understanding of the molecular pathogenesis of genetic heart diseases can drive efforts to develop novel therapeutic agents. Overall, hiPSC-CMs are considered to revolutionise the field, based on their limitless availability, and on the fact that genomic and proteomic analysis in a human context, high throughput screenings, novel drug discovery, and disease modelling open new horizons. They also provide vital information to improve and validate in silico models. To fulfil this promise, however, we first need to overcome the current roadblocks, including immaturity, homogeneity, and the lack of relevant 3D architectures and complex structures.

In summary, the constant development of hiPSC-CMs and protocols carries immense potential in drug testing and drug discovery, disease modelling, and in regenerative therapies. From bench to bedside, clinical-scale production for regenerative medicine is crucial, while solving risks, such as tumorigenicity of undifferentiated iPSCs or proarrhythmic activities of spontaneously active hiPSC-CMs, are equally important. Current trends show that the purity of hiPSC-CMs is key in vivo, however, drug screening and development requires mature hiPSC-CMs in vitro. Creating more complex systems (e.g., 3D cultures instead of 2D, bioreactors, and EHTs) may help to understand their physiology better, achieve greater maturation, and ultimately, connect basic research with clinical studies to completely exploit the translational potential in the field.

## Figures and Tables

**Table 1:** Examples of functional readouts for determining cardiotoxicity in cardiomyocyte cellular models. APA, action potential amplitude; APD, action potential duration; BPM, beats per minute; BR, beat rate; CL, cycle length; ECG, electrocardiogram; FPD, field potential duration; MCS, maximum contraction speed; MRS, maximum relaxation speed; OAP, optical action potential; RMP, resting membrane potential; TdP, torsade de pointes type polymorphic ventricular tachyarrhythmia.

Assay	Method	Measured Parameters	Possible Applications	References
Field potential measurement	Multielectrode array systems	stand-alone FPD sodium spike amplitude RR-interval (beat-to-beat interval) spontaneous BR network analysis (syncytium)	direction and magnitude of depolarisation QT interval, beat-to-beat variability heart rate propagation and contractility	[92,93]
	CardioExcyte 96	FPD, impedance	QT interval on ECG, prediction of TdP risk	[94]
Action potential measurement	CellOPTIQ	depolarisation time and APD by voltage sensitive dye, spontaneous activity	assessing hiPSC-CM function on hydrogels, drug evaluation	[95]
	CardioExcyte 96	myocardial cell activity, BPM, FPD	risk prediction model for TdP in hiPSC-CMs, tool for compound-induced arrhythmias	[94]
	Patch-clamp/ Sharp microelectrode	APD, APA, V_max_, ion currents, RMP	electrophysiological characterisation, drug-induced arrhythmias, sequential pharmacological dissection	[96,97,98]
	µGMEA	APD, APA, V_max_, FPD, RR-interval	long-term electrophysiological recordings, dynamic changes in transmembrane potential of hiPSC-CMs in network, spatial heterogeneity	[99]
	Optical mapping	OAP, CL, d (−F)/dt_max_, APD	detection of propensities for drug-induced tachyarrhythmias	[100]
Calcium measurement	CellOPTIQ	intracellular Ca concentration, Ca transient amplitude, Tau	assessing hiPSC-CM function on hydrogels, drug evaluation	[95]
	Epifluorescence with simultaneous electrophysiology	intracellular Ca concentration, Ca transient amplitude, contractility, Tau, SR content, release kinetics, systolic and diastolic calcium levels	characterisation and drug-induced arrhythmias, Ca flux balance	[101,102,103]
	FLIPR Tetra system	Ca transient peak frequency, amplitude, rise time and decay time	cardiotoxicity assessment of a compound (contractility and arrhythmogenic potential)	[104]
Contractile function	CellOPTIQ	contraction amplitude, duration, relaxation duration	assessing hiPSC-CM function on hydrogels, drug evaluation	[95]
	Cell motion analysis	MCS, MRS, contraction-relaxation duration, BR	detection of drug-induced changes in contractility	[105]
	Single cell contraction measurement	single cell shortening, BR	assessing drug effects	[96]
	Video-based analysis	BR, beating velocity, maximum contraction and relaxation	detection of dysfunctional CM contractility	[106]

## Data Availability

The original contributions presented in the study are included in the article; further inquiries can be directed to the corresponding author/s.

## Abbreviations

AF	atrial fibrillation
AI	artificial intelligence
AP	action potential
APA	action potential amplitude
APD	action potential duration
BPA	bisphenol A
BPAF	bisphenol F
BPM	beats per minute
BR	beat rate
CAST	Cardiac Arrhythmia Suppression Trial
CF	cardiac fibroblast
CiPA	Comprehensive in vitro Proarrhythmia Assay
CL	cycle length
CM	cardiomyocyte
cTnT	cardiac troponin T
CVD	cardiovascular disease
DAPI	4′,6-diamidino-2-phenylindole
DL	deep learning
ECG	electrocardiogram
ECM	extracellular matrix
ECT	engineered cardiac tissues
EHT	engineered heart tissue
EMV	endothelial cell-derived microvesicle
ESC	embryonic stem cell
FDA	US Food and Drug Administration
FPD	field potential duration
GATA4	GATA binding protein 4
hESC-CM	human embryonic stem cell-derived cardiomyocyte
hiPSC	human induced pluripotent stem cell
hiPSC-CM	human induced pluripotent stem cell-derived cardiomyocyte
hiPSC-aCMs	human induced pluripotent stem cell-derived atrial cardiomyocyte
HOC	heart-on-a-chip
HUVEC	human umbilical vein endothelial cells
IDE	interdigitated electrodes
LDH	lactate dehydrogenase
LQTS	long QT syndrome
MCS	maximum contraction speed
MEA	multielectrode array
ML	machine learning
MRS	maximum relaxation speed
NFAT	nuclear factor of activated T-cells
NGS	next-generation sequencing
Nkx2.5	homeobox protein Nkx2.5
OAP	optical action potential
PKC	protein kinase C
RMP	resting membrane potential
ROS	reactive oxygen species
SERCA	sarcopendoplasmic reticulum calcium-ATPase
SK	small conductance calcium-activated potassium channel
SNP	single nucleotide polymorphism
SQTS	short QT syndrome
SR	sarcoplasmic reticulum
SWORD	Survival with oral d-sotalol Trial
TdP	torsade de pointes type polymorphic ventricular tachyarrhythmia
VEGF	vascular endothelial growth factor
